# Towards decision-making using individualized risk estimates for personalized medicine: A systematic review of genomic classifiers of solid tumors

**DOI:** 10.1371/journal.pone.0176388

**Published:** 2017-05-09

**Authors:** Daniel M. Trifiletti, Vanessa N. Sturz, Timothy N. Showalter, Jennifer M. Lobo

**Affiliations:** 1 Department of Radiation Oncology, University of Virginia School of Medicine, Charlottesville, VA, United States of America; 2 Department of Public Health Sciences, University of Virginia School of Medicine, Charlottesville, VA, United States of America; University of North Carolina at Chapel Hill School of Medicine, UNITED STATES

## Abstract

Recent advances in the understanding of the genetic underpinnings of cancer offer the promise to customize cancer treatments to the individual through the use of genomic classifiers (GCs). At present, routine clinical utilization of GCs is uncommon and their current scope and status, in a broad sense, are unknown. As part of a registered review (PROSPERO 2014:CRD42014013371), we systematically reviewed the literature evaluating the utility of commercially available GCs by searching Ovid Medline (PubMed), EMBASE, the Cochrane Database of Systematic Reviews, and CINAHL on September 2, 2014. We excluded articles involving pediatric malignancies, non-solid or non-invasive cancers, hereditary risk of cancer, non-validated GCs, and GCs involving fewer than 3 biomarkers. A total of 3,625 studies were screened, but only 37 met the pre-specified inclusion criteria. Of these, 15 studies evaluated outcomes and clinical utility of GCs through clinical trials, and the remainder through the use of mathematical models. Most studies (29 of 37) were specific to hormone-receptor positive breast cancer, whereas only 4 studies evaluated GCs in non-breast cancer (prostate, colon, and lung cancers). GCs have spurred excitement across disciplines in recent decades. While there are several GCs that have been validated, the general quality of the data are weak. Further research, including prospective validation is needed, particularly in the non-breast cancer GCs.

## Introduction

Over the past 30 years, there have been substantial advances in our knowledge of the genetic underpinnings of cancer. The increase in this knowledge, and in the technology to evaluate it, has generated tremendous excitement because of its potential to customize therapies at the patient-specific level and deliver on the promise of personalized medicine. There is an increasing emphasis on “precision oncology” or “genomics-driven oncology” [1,2], with individualized therapy strategies driven by molecular “-omics” information.

A genomic classifier (GC) offers the opportunity to select patients most likely to respond to therapy, based on stratification of probability of a clinical outcome according to a DNA or RNA expression signature [3,4]. This provides the potential to intensify therapy in patients with high-risk disease, improving cure rates, and avoid the ‘overtreatment’ of patients with biologically low-risk disease that historical, clinical, or histopathologic criteria cannot otherwise distinguish. Since the mid-2000s, several commercially available breast cancer GCs have been approved for coverage by Medicare & Medicaid [5]. Population-based research has identified increasing utilization rates of GCs among breast cancer patients, with concordant reduction in the proportion of women with hormone receptor positive cancer receiving chemotherapy [6]. Recent series estimate that 18% of women with breast cancer in the U.S. undergo the 21-gene recurrence score assay, which is only one of many [7]. Comparatively, there has been surprisingly little clinical implementation of GCs for other solid tumors.

Additional research is needed to deliver on the promise of GCs for solid tumors [2,8]. Despite the promise of genomics-driven cancer medicine, its clinical implementation is limited by a relative lack of prospective evidence regarding genomic assay validation and clinical performance [9]. The availability of strong evidence from well-designed, prospective trials is a significant challenge and rate-limiting step in the development of GCs [3].

Our purpose was to describe the current state of GCs and delineate areas of research that could validate their routine use in clinic. We systematically review and report the current evidence evaluating the utility of commercially available GCs for solid tumors of adults. Our study describes the outcomes and clinical utility measure of GCs as studied through clinical trials or the use of mathematical models.

## Methods and materials

**Fig 1:**
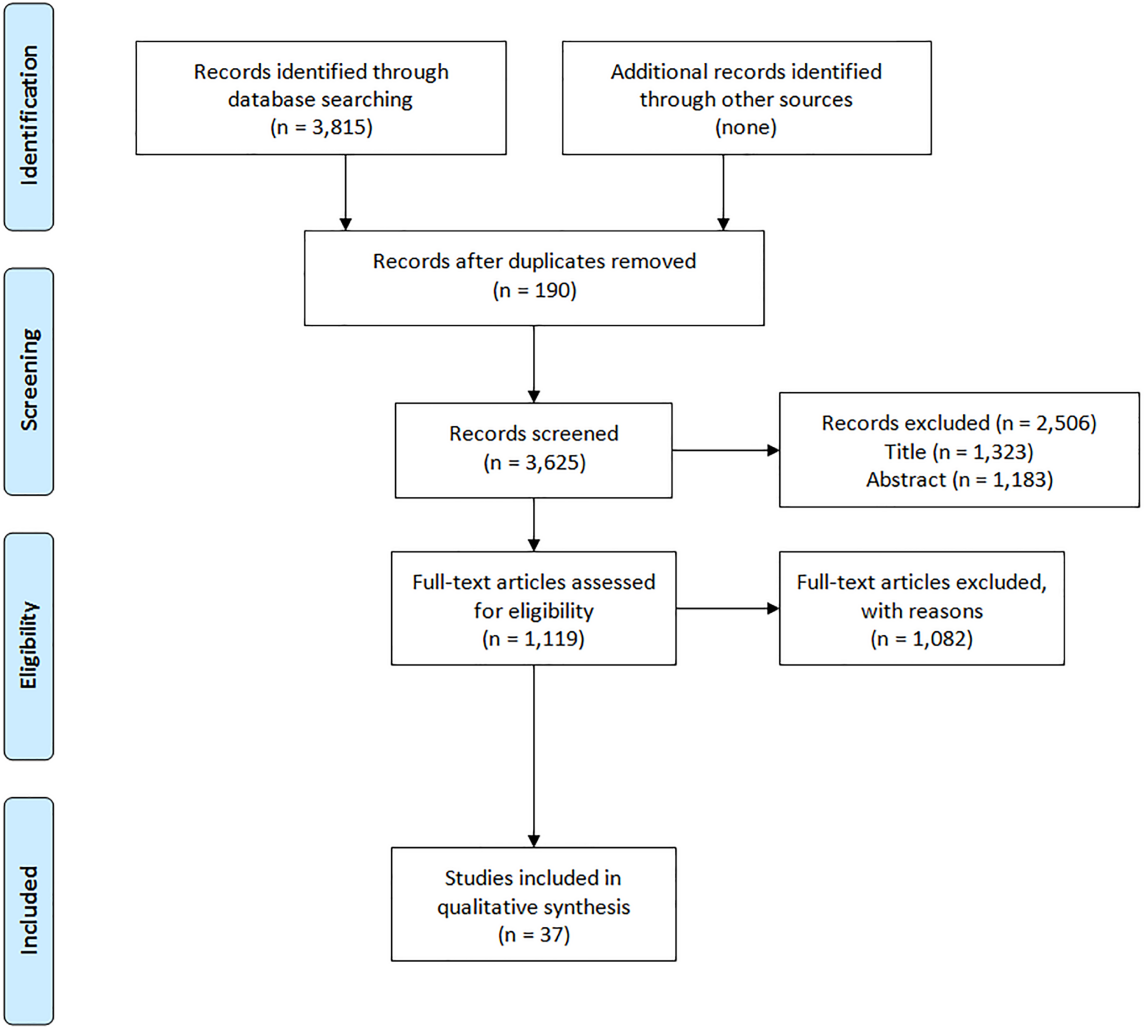
PRISMA flow diagram [10].

As part of a registered, PROSPERO International prospective systematic review (PROSPERO 2014:CRD42014013371), we conducted literature database searches of Ovid Medline (PubMed), EMBASE, the Cochrane Database of Systematic Reviews, and CINAHL on September 2, 2014. The MeSH search criteria are provided in the supporting information (S1 File), but generally includes terms associated with genomic and/or personalized cancer care. We restricted search criteria those reported in English. This resulted in 3,815 articles with 190 duplicates (3,625 unique articles, Fig 1). The PRISMA checklist is provided in the supporting information (S2 File).

**Fig 2:**
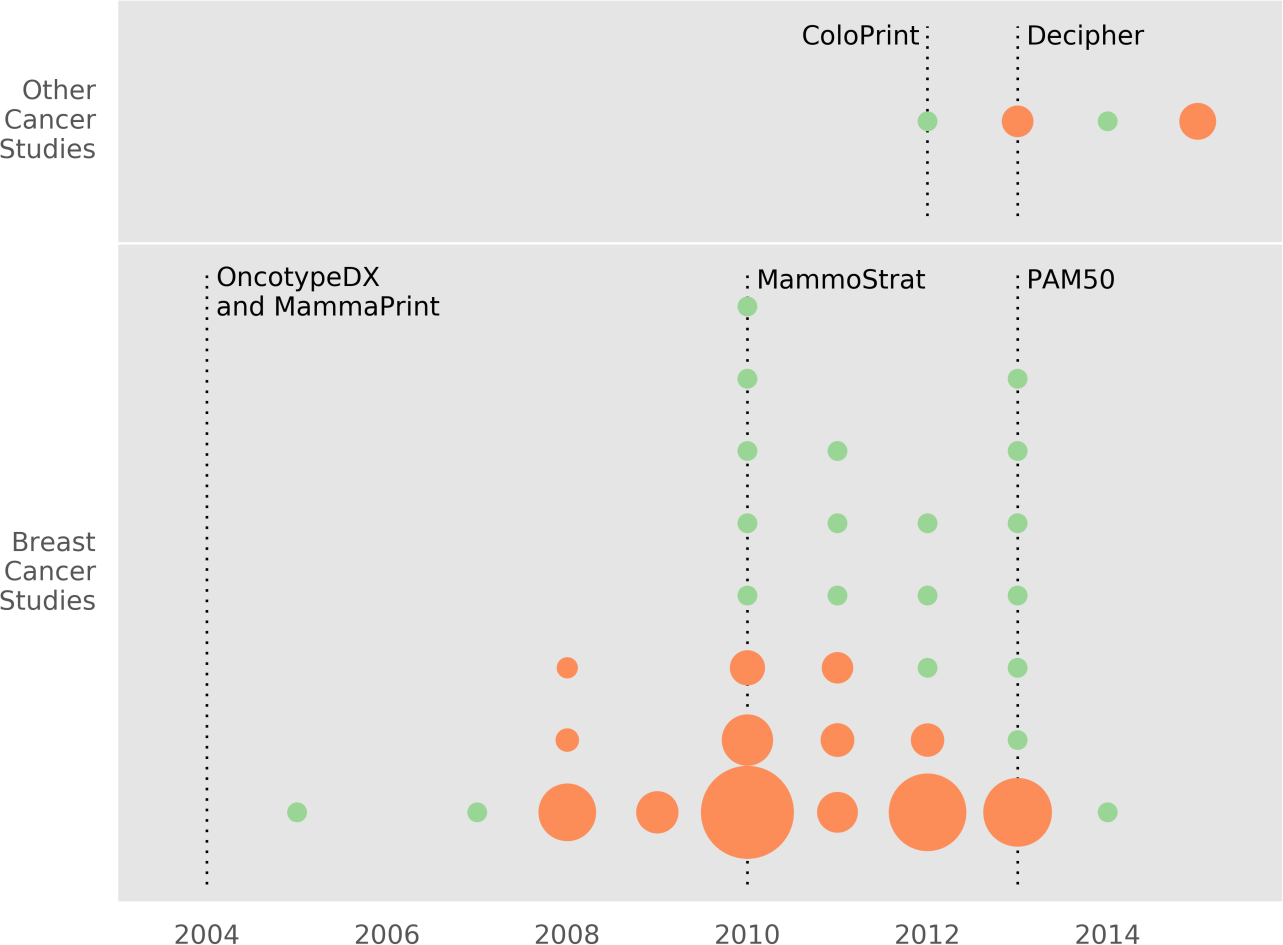
Timeline of the publication of the studies and the date relevant GCs became commercially available [12–17]. Each dot represents a study, with green dots for modeling studies and orange dots for clinical studies. The dot diameter for clinical studies corresponds to the number of patients in the study.

Two investigators independently reviewed manuscript titles and abstracts to identify original data studies that involved the use of validated GCs to demonstrate clinical utility. Clinical utility is demonstrated when the test is shown to improve clinical outcomes and/or alter clinical decisions. Studies were required to involve solid tumors, adult patients (≥ 18 years old), and GCs with 3 or more biomarkers. Manuscripts involving pediatric malignancies, non-solid or non-invasive tumors (e.g., leukemia, ductal carcinoma in situ, etc.), hereditary risk of cancer, non-validated GCs, and GCs involving less than 3 biomarkers were excluded (Fig 1). In addition, manuscripts were reviewed independently by the two investigators for quality by applying the general principles of the Reporting Recommendations for Tumor Marker Prognostic Studies (REMARK) checklist items [11]. A third investigator served to resolve all coding disagreements. Each included manuscript was assessed for clinical site, assay(s) used, number of patients or simulated patients, the specific clinical population the results apply to, the methodology, the main contribution of the study, and the country of origin. Data extraction was completed using a pre-defined spreadsheet; one investigator performed the data extraction while a second investigator reviewed the spreadsheet to confirm correct data extraction. We present a figure to show the timeline of when the studies were published, number of patient specimens included in each clinical study (shown by dot size), and when the major GCs were available commercially (Fig 2).

## Results

We identified 3,625 manuscripts for title review according to the above methods. As shown in Fig 1, this was reduced to 2,302 manuscripts for abstract review and 1,119 studies for full text review. After this final review, 37 manuscripts were included [18–54]. A total of 273 abstracts and 55 manuscripts needed a third investigator to resolve coding disagreements. Tables 1 and 2 depict the key characteristics of each included study. Table 1 provides a summary of the breast cancer studies while Table 2 presents studies for all other types of cancer. Of the 37 studies, 15 studies evaluated outcomes and clinical utility of GCs through clinical trials, and the remainder through the use of mathematical models.

**Table 1:** Papers evaluating breast carcinoma.

Year	Site	Assay	n	Population	Methodology	Main Conclusion	Country
*Clinical Outcomes*							
2008	Breast	Oncotype DX	72	HR+, locally advanced breast cancer	Retrospective analysis of clinical outcomes	GC predicts pathologic complete response to neoadjuvant chemotherapy	USA [18]
2008	Breast	Oncotype DX	465	HR+ breast cancer	Case control study	GC predicts cancer control/survival	USA [19]
2008	Breast	Oncotype DX	58	HR+, early stage breast cancer	Retrospective analysis of clinical outcomes	GC affects adjuvant therapy decision making	USA [20]
2009	Breast	Oncotype DX, 78-gene profile, Two-Gene-Index	246	HR+, early stage breast cancer	Retrospective analysis of clinical outcomes	GC predicts cancer control/survival	Netherlands [21]
2010	Breast	Oncotype DX	367	HR+, node-positive, postmenopausal breast cancer	Retrospective analysis of clinical outcomes	GC predicts cancer control/survival	USA [22]
2010	Breast	Oncotype DX	1,231	HR+, postmenopausal breast cancer	Retrospective analysis of clinical outcomes	GC predicts cancer control/survival	UK [23]
2010	Breast	MammaPrint	168	HER2+, early stage breast cancer	Retrospective analysis of clinical outcomes	GC predicts cancer control/survival	Netherlands [24]
2011	Breast	MammaPrint, Oncotype DX, 76-gene signature	228	Breast cancer	Retrospective analysis of clinical outcomes	Each GC performed similarly	USA, Japan, and Italy [25]
2011	Breast	Oncotype DX	154	HR+, early stage breast cancer	Prospective GC vs. expert opinion	Experts tend to overestimate risk of recurrence compared to GC	USA [26]
2011	Breast	Oncotype DX	133	Breast cancer	Retrospective analysis of clinical outcomes	GC predicts cancer control/survival among ER+ tumors	USA [27]
2012	Breast	PAM50, Oncotype DX	151	HR+, node negative breast cancer	Retrospective analysis of clinical outcomes	Each GC agreed except in low risk patients	USA [28]
2012	Breast	Oncotype DX	853	HR+, early stage breast cancer	Retrospective analysis of clinical outcomes	GC less utilized among African Americans and demonstrated higher recurrence scores	USA [29]
2013	Breast	Oncotype DX	665	HR+, early stage breast cancer	Retrospective analysis of clinical outcomes	GC predicts cancer control/survival	USA [30]
*Modeled Outcomes*							
2005	Breast	Oncotype DX	100	HR+, node-negative breast cancer	Cost-effectiveness, Markov Model	GC is cost effective	USA [31]
2007	Breast	Oncotype DX	688	HR+, early stage breast cancer	Cost-effectiveness, Markov Model	GC is cost effective	USA [32]
2010	Breast	MammaPrint	427	Early stage breast cancer	Cost-effectiveness, Markov Model	GC is cost effective	USA [33]
2010	Breast	Oncotype DX	368	HR+, early stage breast cancer	Cost-effectiveness, Markov Model	GC is cost effective	Israel and USA [34]
2010	Breast	Oncotype DX	89	HR+, early stage breast cancer	Prospective pre/post GC decision making	GC affects adjuvant therapy decision making	USA [35]
2010	Breast	MammaPrint	305	HR+, node negative breast cancer	Cost-effectiveness, Markov Model	GC is cost effective	Netherlands and Austria [36]
2010	Breast	Oncotype DX	-	HR+, HER2-,early stage breast cancer	Cost-effectiveness, Markov Model	GC is cost effective	Canada [37]
2011	Breast	Oncotype DX	925	HR+, node-negative breast cancer	Cost-effectiveness, Markov Model	GC is cost effective	USA [38]
2011	Breast	Oncotype DX	2,000,000	HR+, HER2-,early stage breast cancer	Cost-effectiveness, Markov Model	GC is cost effective	USA [39]
2012	Breast	Oncotype DX	-	HR+, node-positive breast cancer	Cost-effectiveness, Modified Markov Model	GC is cost effective	UK [40]
2012	Breast	Oncotype DX	489	HR+, node-negative breast cancer	Cost-effectiveness, Markov Model	GC is cost effective	Canada [41]
2012	Breast	Oncotype DX	1,000	HR+ breast cancer	Cost-effectiveness, Markov Model	GC is cost effective	Canada [42]
2012	Breast	Oncotype DX, MammaPrint	-	HR+, early stage breast cancer	Cost-effectiveness, Markov Model	GC is cost effective	USA [43]
2013	Breast	Oncotype DX	151	HR+, HER2-, 0–3 nodes, breast cancer	Prospective pre/post GC decision making	GC affects adjuvant therapy decision making	Australia [44]
2013	Breast	Oncotype DX	142	HR+, node-negative breast cancer	Prospective pre/post GC decision making	GC affects adjuvant therapy decision making and is cost effective	UK impact, decision [45]
2013	Breast	Oncotype DX	1,000	HR+, HER2-,early stage breast cancer	Cost-effectiveness, Markov Model	GC is cost effective	Canada [46]
2013	Breast	Oncotype DX	-	HR+, early stage breast cancer	Cost-effectiveness, Markov Model	GC is cost effective	USA [47]
2013	Breast	MammaPrint	427	HR+, early stage breast cancer	Cost-effectiveness, Markov Model	GC is cost effective	Netherlands and Austria [48]
2013	Breast	Oncotype DX, IHC4, MammaPrint and Mammostrat	-	HR+, HER2-,early stage breast cancer	Systematic review of cost effectiveness	GC is cost effective	Multiple [49]
2014	Breast	Mammostrat	-	HR+, early stage breast cancer	Cost-effectiveness, Markov Model	GC is cost effective	USA and UK [50]

Summary of papers included in this analysis evaluating breast carcinoma. Abbreviations: HR, hormone receptor; GC, genomic classifier.

**Table 2:** Papers evaluating non-breast carcinoma.

Year	Site	Assay	n	Population	Methodology	Main Conclusion	Country
*Clinical Outcomes*							
2013	Colon	ColoPrint	135	Stage II colon cancer after resection	Retrospective analysis of clinical outcomes	GC predicts cancer control/survival	Germany [51]
2015^a^	Prostate	CAPRA-S, Decipher	185	High risk prostate cancer after radical prostatectomy	Retrospective analysis of clinical outcomes	GC predicts cancer control/survival	USA [52]
*Modeled Outcomes*							
2012	Colon	12-gene assay	-	Stage II colon cancer after resection	Cost-effectiveness, Markov Model	GC is cost effective	USA [53]
2014	Lung	14-gene assay	433	Early stage non-small cell lung cancer after resection	Cost-effectiveness, Markov Model	GC is cost effective	USA [54]

Summary of papers included in this analysis evaluating non-breast carcinoma. Abbreviations: GC, genomic classifier.

^a^Note: While the manuscript publication year is 2015, it was initially published online July 2, 2014; thus, this manuscript was published during our search period.

Fig 2 depicts the timeline of the publication of the studies and the date relevant GCs became commercially available [12–17]. Each dot represents a study, with green dots for modeling studies and orange dots for clinical studies. The dot diameter for clinical studies corresponds to the number of patients in the study. In general, breast cancer GCs were developed and commercially available earlier than GCs for other cancers.

### Breast cancer

Thirty-three (89%) of studies evaluated breast cancer, and of these, 29 (89%) were specific to hormone-receptor positive breast cancer, and 31 (94%) concerned the Oncotype DX^®^ GC (Table 1). Among the trials concerning breast cancer GCs, 13 (39%) concern the clinical validation of GCs, mostly through the testing of prospectively collected tissue banks and evaluation of various clinical outcomes (overall survival, cancer recurrence, pathologic response to neoadjuvant therapy, etc.).

Two studies presented comparisons of multiple GCs. Iwamoto et al. compared six distinct assays for breast cancer (MammaPrint, Oncotype DX^®^, a 76-gene signature assay, mitotic kinase prognostic score, MKI67 mRNA expression, and molecular subtype). They demonstrated that the assays generally performed similarly in their abilities to predict 5-year overall survival, progression-free survival, and pathologic complete response [25]. Kelly et al. compared the Oncotype DX^®^ GC to the PAM50 Breast Cancer Intrinsic Classifier^TM^ and demonstrated general agreement between the two [28].

Of the breast cancer GC articles, 20 (61%) are based in mathematical models and generally concern cost-effectiveness. The main type of mathematical model used is a Markov model, a state-transition model used to simulate the health outcomes and costs for a cohort of patients. Each article included demonstrated that the use of GCs in breast cancer was cost-effective in a variety of reimbursement models (Table 1). In addition, 4 articles demonstrated that the use of GCs in breast cancer altered decisions regarding the recommendation for or against adjuvant therapy.

### Non-breast cancer

As demonstrated in Table 2, 4 studies (11%) evaluated GCs in non-breast cancer. Hornberger et al. demonstrated that the ColoPrint GC predicted clinical outcomes in stage II colon cancer [53], and Maak et al. went on to demonstrate its cost-effectiveness in this setting [51]. Cooperberg et al. provided retrospective clinical evidence supporting the Decipher GC [52]. While Roth et al. demonstrated the cost-effectiveness of a 14-gene GC for early stage non-small cell lung cancer following surgery [54], other articles regarding this classifier did not meet our pre-defined inclusion criteria.

## Discussion

Our results provide a summative analysis of the current state of the clinical research supporting the validation of GCs in patients with some solid tumors. While there are several commercially available GCs, the bulk of the existing published data are evaluations of breast cancer GCs.

While breast cancer is a common malignancy that usually requires multimodality therapy, cure rates for most women with breast cancer is already high. Regardless, there is a subset of patients with breast cancer that go on to die from their disease, and GCs are poised to identify these patients and potentially cure them. The development of GCs regarding more commonly fatal diseases such as locally advanced lung cancer or glioblastoma multiforme may have limited clinical utility, since most patients with poor-prognosis cancers will receive the most intensively validated therapy and a decision aid may not be clinically relevant for personalized decisions.

Interestingly, all of the articles in this systematic review regard the decision for (or against) adjuvant chemotherapy following definitive surgical resection. For most cancers however, there are multiple therapies that could be informed through GCs. In head and neck cancer, for example, many patients undergo definitive surgical resection and adjuvant therapy while others are receive definitive chemoradiotherapy (without surgery) without clear existing evidence as to which (if either) improves outcomes for patients. As another example, it is unlikely that the superiority (or inferiority) of radical prostatectomy over radiotherapy will ever be established, but it is possible that GCs could serve to define a subset of patients that would be better served with either therapy.

GCs have positioned themselves in a gap in cancer care that has obsessed researchers for decades. On one side are diseases that have targetable, gene-specific mutations (e.g., ALK-rearranged non-small cell lung cancer) and on the other side are markedly heterogeneous diseases where only non-discriminatory therapies have effect. GCs have the ability to fill this gap by analyzing numerous genes and weighting them based on their ability to drive cancer recurrence and metastasis, keying physicians in that more intensive or alternative therapy is warranted.

There are several trends across GCs that should be noted including that the majority of patients included in these studies are Caucasian. Baseline genetic heterogeneity between racial groups could have an impact on the external validity of these tests, and further research in this area is needed before broad application of any genetic test is appropriate across a diverse population. Finally, relatively few of the studies included comparisons of multiple GCs; as noted by Hunter [55], more research is needed to compare how risk categorizations differ between GCs.

## Conclusion

GCs promise an era of precise, personalized cancer care. While there are several GCs that have been accepted for clinical use (particularly in breast cancer), our review demonstrates that there are a relatively limited number of studies available to provide supportive evidence of clinical utility. We await the prospective validation of several of the alternative GCs for other solid tumors. Further research, including prospective validation is needed, particularly for non-breast cancer GCs.

## Supporting information

S1 File MeSH search criteria. (PDF)

S2 File PRISMA 2009 checklist. (DOC)
