# Supplementary material for: Towards decision-making using individualized risk estimates for personalized medicine: A systematic review of genomic classifiers of solid tumors
Source: PLoS One. 2017 May 9;12(5):e0176388. doi: 10.1371/journal.pone.0176388 (PMC5423583; doi:10.1371/journal.pone.0176388)
Supplement: S1 File — (PDF) [file pone.0176388.s001.pdf]

S1 File. MeSH search criteria.

Purpose: probability[MeSH Terms] OR "risk stratification"[All Fields] OR "risk assessment"[All Fields] OR "risk adjustment"[All Fields] OR "recurrence score"[All Fields] OR "precision medicine"[All Fields] OR "personalized medicine"[All Fields] OR "personalised medicine"[All Fields] OR "individualized medicine"[Mesh] OR "clinical decision making"[All Fields] OR "genomics-driven medicine"[All Fields]

AND

Type: "genetic techniques"[Majr] OR "proteomics"[MeSH Terms] OR "pharmacogenetics"[Mesh] OR "metabolomics"[MeSH Terms] OR biological assay[MeSH Terms] OR "gene expression profiling"[All Fields] OR "transcriptome profiling"[All Fields] OR "next generation sequencing"[All Fields] OR "whole genome sequencing"[All Fields] OR "whole exome sequencing"[All Fields] OR "whole transcriptome sequencing"[All Fields] OR "targeted genome sequencing"[All Fields] OR "RNA sequencing"[All Fields] OR "genome copy number variation"[All Fields] OR "comparative genomic hybridization"[All Fields] OR "DNA methylation assays"[All Fields] OR "chromatin immunoprecipitation assay"[All Fields] OR "ChIP assay"[All Fields] OR "bisulfite sequencing"[All Fields] OR "gene expression microarray"[All Fields] OR “biological assay” [All Fields] OR “prognostic assay” [All fields] OR "genomic classifier"[All Fields] OR "validated prognosticator"[All Fields] OR multiplex [All Fields]

AND

Cancer: neoplasms[MeSH Terms] OR neoplasm[All Fields] OR Cancer[TIAB]

AND

Methods: "cost-benefit analysis"[MeSH Terms] OR "cost-benefit analysis"[All Fields] OR "health care costs"[MeSH Terms] OR "health care costs"[All Fields] OR "cost effectiveness"[All Fields] OR "cost utility"[All Fields] OR "quality of life"[MeSH Terms] OR "quality of life"[All Fields] OR "quality-adjusted life years"[MeSH Terms] OR "quality adjusted life years"[All Fields] OR Markov[All Fields] OR "Outcome Assessment (Health Care)"[Mesh] OR "outcome assessment"[All Fields] OR "models, statistical"[MeSH Terms] OR model [All Fields] OR "computer simulation"[Mesh] OR simulation[All Fields] OR "regression model"[All Fields] OR “chi square test”[All Fields] OR “quality-adjusted life expectancy”[All Fields]

AND

Language: English[lang]

AND

Subjects: Humans[MeSH]
